# SARS-CoV 2 Infection (Covid-19) and Cardiovascular Disease in Africa: Health Care and Socio-Economic Implications

**DOI:** 10.5334/gh.829

**Published:** 2021-03-15

**Authors:** Okechukwu S. Ogah, Ejiroghene M. Umuerri, Adewole Adebiyi, Olanike A. Orimolade, Mahmoud U. Sani, Dike B. Ojji, Amam C. Mbakwem, Simon Stewart, Karen Sliwa

**Affiliations:** 1 Department of Medicine, Faculty of Clinical Sciences, College of Medicine, University of Ibadan, Nigeria/Department of Medicine, University College Hospital Ibadan, NG; 2 Institute of Advanced Medical Research and Training, College of Medicine, University of Ibadan, NG; 3 Department of Medicine, Delta State University, Abraka, Delta State Nigeria/Department of Medicine, Delta State University Teaching Hospital, Oghara, Delta State, NG; 4 Department of Medicine Bayero University Kano & Aminu Kano University Teaching Hospital, Kano, NG; 5 Department of Medicine, University of Abuja, Abuja, Nigeria/Department of Medicine, University of Abuja Teaching Hospital, Abuja, NG; 6 Department of Medicine, University of Lagos, Akoka, Lagos, Nigeria/Department of Medicine, Lagos University Teaching Hospital, Idi-araba, Lagos, NG; 7 Torrens University Australia, Adelaide, South Australia, AU; 8 Hatter Institute for Cardiovascular Research in Africa, University of Cape Town, Cape Town, ZA

**Keywords:** SARS CoV-2, Covid-19, Heart disease, Cardiac disease, Cardiovascular disease, Heart failure, Myocarditis, Arrhythmias, health system, socio-economic, Africa

## Abstract

The current pandemic of SARS-COV 2 infection (Covid-19) is challenging health systems and communities worldwide. At the individual level, the main biological system involved in Covid-19 is the respiratory system. Respiratory complications range from mild flu-like illness symptoms to a fatal respiratory distress syndrome or a severe and fulminant pneumonia. Critically, the presence of a pre-existing cardiovascular disease or its risk factors, such as hypertension or type II diabetes mellitus, increases the chance of having severe complications (including death) if infected by the virus. In addition, the infection can worsen an existing cardiovascular disease or precipitate new ones.

This paper presents a contemporary review of cardiovascular complications of Covid-19. It also specifically examines the impact of the disease on those already vulnerable and on the poorly resourced health systems of Africa as well as the potential broader consequences on the socio-economic health of this region.

## Introduction

The first reported case of the infection caused by the Novel Coronavirus, initially called nCoV 2019, occurred on December 8, 2019 in Wuhan City, Hubei Province, China. The virus was later renamed Severe Acute Respiratory Syndrome Coronavirus-2 (SARS CoV-2), and the disease called Coronavirus Disease 2019 (Covid-19). Since then, the infection crossed many geographic boundaries, and it is now a global pandemic. It was initially reported to the World Health Organization (WHO) on December 31, 2019, declared a global health emergency on January 30, 2020, and considered to be a pandemic on March 11, 2020. SARS CoV-2 infection is characterized by high infectivity and rapid transmission as well as possibility of transmission during an asymptomatic period.

The main system involved in Covid-19 is the respiratory system [1,2]. Respiratory complications range from a mild flu-like illness symptoms to a fatal respiratory distress syndrome or a severe and fulminant pneumonia. However, the presence of pre-existing cardiovascular disease or its risk factors such as hypertension or type II diabetes mellitus increases the chances of individuals having severe forms of the disease [3]. Moreover, the infection can worsen a pre-existing cardiovascular disease or precipitate new ones. In a recent modelling study, Clark et al. [4] reported that about a fifth of persons worldwide may be at increased risk of severe Covid-19 infection. These are individuals who may be infected because they have pre-existing co-morbidities. The risk varies with age. It was estimated that 6% of males to be at high risk compared with 3% of females. The populations with increased risk include countries with elderly populations, those with a high burden of HIV and AIDs, such as countries in Africa, and countries with a high burden of diabetes mellitus, such as small island nations. It was estimated that the number of individuals at increased risk were most sensitive to the prevalence of chronic kidney disease, diabetes, cardiovascular disease, and chronic respiratory disease.

For many high-income regions of the world, factors such as their age and risk-factor profile (including high obesity rates) of local populations will determine the relative impact of the disease. However, in low-to-middle-income regions, the pattern of disease and its impact are likely to be different. Accordingly, the emergence of this highly infectious and devastating disease is now a major challenge to the global health system and, indeed, the global economy. For the approximate 800 million mainly impoverished people living in sub-Saharan Africa, cardiovascular disease (both in the form of heart and cerebrovascular disease) has emerged as a growing public health problem [5].

Already poorly resourced health systems and fragile economies are being challenged by a growing number of affected cases; with many more women and younger individuals being affected than in high-income countries. The added complication/burden of the Covid-19 pandemic is potentially even more devastating in this context.

The biology of SARS-CoV-2, its life cycle, environmental sensitivity, mode of transmission, epidemiology and patient characteristics have been reviewed extensively elsewhere [6,7,8,9,10,11,12,13,14,15,16,17,18,19,20,21]. Therefore, the main thrust of this paper is to review the existing knowledge of the cardiovascular complications associated with Covid-19. We reviewed literature extracted from PubMed and other major databases, original papers, review articles, editorials, opinions, commentaries, correspondences and statements/recommendations by major cardiac societies from December 2019 through 30 April 2020. This is a narrative review and no systematic search for relevant literature was performed. We then consider and discuss the profound impact of the Covid-19 pandemic on the health systems and economies of sub-Saharan Africa.

## Pathogenesis of SARS-CoV-2 induced cardiovascular injury

As noted, the principal symptoms and complications of Covid-19 is mainly respiratory, which is consistent with the primary infection. However, as also noted, other systemic complications, including the cardiovascular system. In one report, about 19% of hospitalized cases have evidence of cardiac injury. This is said to be associated with a higher risk of mortality [22,23,24]. Covid-19 is able to infect its human host via the angiotensin converting enzyme-2 (ACE-2) receptor [11,25]. ACE-2 is a membrane-bound protein expressed in many human cells such as the vascular endothelium, cardiovascular tissues, intestinal epithelia and renal tissues [26]. The respiratory tract is the primary target of the infection, although other systems can also be involved [26]. The cardiovascular system’s involvement may be through one or more of the following mechanisms; direct myocardial damage, systemic inflammation, plaque rupture and thrombosis of the coronary arteries, alteration in myocardial oxygen demand and supply ratio, hypoxemia electrolyte perturbations, dysautonomia and side effects of various treatments.

- Direct myocardial injury: The binding of the virus to ACE-2 receptor can bring about alteration in the ACE-2 signaling pathways. This will lead to acute lung and myocardial injury [27,28].
- Systemic inflammation: SARS-CoV-2 infection is characterized by acute systemic inflammation. High circulating inflammatory response and ‘cytokine storm’ has been observed in individuals with severe disease [29].
- Alteration in myocardial oxygen demand and supply ratio: Acute rise in myocardial oxygen demand due to systemic infection and hypoxia from respiratory disease will in turn lead to myocardial oxygen demand and supply mismatch.
- Plaque rupture and acute coronary syndrome: Systemic inflammation as well as increased coronary blood flow (which will increase shear stress) can lead to plaque rupture and acute coronary event. The systemic inflammation may also precipitate a pro-thrombotic state.
- Electrolyte derangements: Electrolyte derangements can result from the systemic illness and this in turn can trigger arrhythmias. The major concern is for serum potassium because of the interaction of SARS-CoV-2 with the RAAS [29].
- Dysautonomia from intense stress may result in stress induced cardiomyopathy.
- Adverse effect of treatment: Chloroquine (CQ), hydroxychloroquine (HCQ), corticosteroids and anti-viral medications do have adverse effects on the cardiovascular system.

## Pathophysiology of SARS-CoV-2 induced cardiovascular injury

The pathophysiological changes in a Covid-19 infection are seen mostly in the lungs with evidence of marked systemic inflammation and effects on the lymphocytes. The virus causes direct damage to the pneumocytes through a viral cytopathic effect. There is also diffuse alveolar damage from acute respiratory distress syndrome (ARDS) [30]. There is an associated marked inflammatory response which leads to multiple organ failure and disseminated intravascular coagulation in some patients [31].

Studies have also shown that markers of inflammation such as IL-6, CRP, IFN-γ and TNF-α are increased in patients infected with SARS-CoV-2. This is said to contribute to the inflammatory response and cytokine storm [32,33]. It is also thought that the viral particle can invade lymphocytes leading to their destruction and lymphopaenia. Finally, severe pneumonia and ARDS seen in patients leads to hypoxia, further end organ damage and death [34].

## Information gleaned from previous viral infections

Much of information learned from the SARS-CoV-2 pandemic stems from lessons gleaned from previous epidemics such as SARS-CoV, MERS-CoV and HIN1 influenza syndrome [22,23,29,35,36,37,38,39,40,41,42,43,44]. In these previous epidemics, it was noted that co-existing cardiovascular disease (CVD) with or without myocardial injury was associated with higher mortality [45] (Table 1).

**Table 1:** Cardiac complications of previous and current coronavirus epidemics [22,23,29,35,36,37,38,39,40,41,42,43,44].

Complications	SARS-CoV(%)	MERS-CoV(%)	H1N1(%)	SARS-CoV-2

Acute heart failure	0.72	–	–	23
Acute cardiac injury	–	–	–	7.2
Tachycardia	46	–	–	1
Bradycardia	14.9	–	–	–
Chest pain	10.4	7–44	5	3.4
Bundle branch block	15.2	–	–	–
Reduced LV function	30.2	–	–	–
Hypotension	50.4	–	–	1
Arrhythmias	–	15.7	–	16.7
Cardiomegaly	10.7	53	–	–
Pericarditis	–	11.1	–	–
SVT	–	11.1	–	–
VT	–	11.1	–	–
VF	–	–	–	–
VT/VF		–	–	5.9
ST-Elevation on 12-lead ECG	–	–	–	0.7

LV= Left Ventricle, SVT= Supraventricular tachycardia, VT= Ventricular tachycardia, VF = Ventricular fibrillation, ECG= Electrocardiogram, SARS= severe acute respiratory syndrome, SARS-CoV= severe acute respiratory syndrome-coronavirus, MERS-CoV= Middle East respiratory syndrome coronavirus.

For instance, SARS-CoV was found to be associated with cardiomyopathy [46]. Other cardiovascular findings include hypotension, arrhythmias and sudden death [27]. Persistent tachycardia was the commonest arrhythmia, which was found in 72% of cases and may persist for weeks. The tachycardia may be due to changing autonomic tone, cardiopulmonary de-conditioning from prolonged bed rest or due to the drug treatment (e.g., corticosteroids) [47]. Other possible causes of tachycardia include impaired pulmonary function, impaired cardiac function, cardiac arrhythmia, thyroid dysfunction, anaemia, autonomic dysfunction and anxiety states [27]. Bradycardia can also occur, but this is transient and was seen in 14.9% of cases [27]. Reversible cardiomegaly without heart failure was seen in 10.7%. Transient atrial fibrillation is an uncommon feature [27]. The mechanism of sudden cardiac death in SARS include; (i) lung injury, hypoxemia and cardiac electrical instability, (ii) myocardial cells and conduction system damage and (iii) extreme anxiety causing endogenous catecholamine release which can lead to electrical instability [47].

In terms of MERS-CoV infection, the mortality is higher than that of SARS-CoV, the early mortality rate being 22–76% compared to 3.6–13%% for SARS-CoV (See Table 2) [48,49].

**Table 2:** Intra-hospital mortality in previous and current coronavirus epidemic.

Coronavirus	Author	Year	Country	Number of subjects	Intra-hospital mortality

**SARS-CoV(%)**	Lee [35]	2003	Hong Kong	138	3.6
	Booth [36]	2003	Canada	144	6.5
	Li [37]	2003	Hong Kong	46	13
	Yu [38]	2006	Hong Kong	121	NR
**MERS-CoV(%)**	Saad [39]	2014	Saudi Arabia	70	60
	Al-Tawfiq [40]	2014	Saudi Arabia	17	76
	Assiri [41]	2013	Saudi Arabia	47	60
	Al-Albdallat [110]	2014	Jordan	9	22
**H1N1 Influenza**	Schoen [42]	2019	Brazil	160	0
**SARS-CoV-2**	Huang [43]	2020	China	41	15
	Wang [23]	2020	China	118	4.3
	Shi [22]	2020	China	416	13.7
	Zhou [29]	2020	China	191	28.2
	Guo [56]	2020	China	187	23

MERS-CoV related infection was common in patients with cardiovascular disease. Common complications include renal impairment (40.9%), rhythm disorders (15.7%) and liver dysfunction (31.4%). Other features include pericarditis and myocarditis [39].

Influenza infection has been shown to exacerbate many forms of cardiovascular disease. It is associated with myocarditis, myocardial infarction and aggravation of heart failure [50]. It has been shown that patients are more likely to have ventricular arrhythmia during seasonal influenza infection [51,52]. Some of the plausible mechanisms include severe systemic inflammatory reactions. The infection is known to precipitate heart failure and related admissions [52]. It also leads to increased cardiac work load and precipitate acute coronary and arrhythmic events [51]. Seasonal influenza infection is also associated with increased cardiovascular related deaths [50,53]. Alternatively, annual an annual influenza vaccination is associated with reduction in all-cause and cardiovascular-related mortality by 18% [54]. Influenza infection causes hypoxia, increased adrenergic activity and increased metabolic workload. It can also lead to hypercoagulable states and consequences such as acute coronary syndrome [54].

## SARS –CoV-2 Infection (Covid-19)

The infection is highly contagious, but the mortality is lower. Acute cardiac injury, shock and arrhythmias are the common cardiac manifestations. This has been shown to occur in 7.2%, 8.7% and 16.7%, respectively [55]. This tends to be higher in those requiring critical care. Elevated cardiac enzymes have been reported in this group [55] (Table 2). Compared to patients with normal troponin levels, those with elevated troponins are at higher risk of acute respiratory distress syndrome, malignant ventricular arrhythmias, acute coagulopathy and acute renal failure [23]. However in another report, elevated troponin is not associated with increased mortality in a multivariate analysis [56]. Acute myocarditis as well as ventricular arrhythmias might be the first mode of presentation of the SARS-CoV 2 infection [24,57]. Myocardial injury may result in atrial or ventricular fibrosis after discharge of Covid-19 patients, and this may form a nidus or substrate for arrhythmia generation. A cardiac MRI may be a useful tool for assessing category of patients with myocardial injury and for subsequent risk stratification [58]. However, this facility is not readily available in sub-Saharan Africa, apart from the issue of cost and personnel. Another issue is that a patient with pre-existing cardiovascular disease may have problems with early diagnosis because symptoms like fatigue, dyspnea and cough are also early symptoms of the Covid-19 infection [59].

Some of the theories postulated on the effects of Covid-19 infection and cardiovascular disease include cardiovascular instability occasioned by the imbalance between the infection induced increase in metabolic demand and reduced cardiac reserve [23]. This is in addition to the inflammatory response and myocardial injury which can increase the risk of acute coronary syndromes and arrhythmias. Another mechanism could be due to severe down-regulation of myocardial and pulmonary ACE-2 pathways resulting in myocardial inflammation, pulmonary oedema and respiratory failure [60]. Since ACE-2 is well expressed in the lungs and cardiovascular system, ACE-2 related signaling pathways might play a role in the cardiac injury. Furthermore, ‘a cytokine storm triggered by an imbalance response by the type 1 and 2 helper – T cells’ may also have a role [43]. There is also the possibility that interferon-mediated immunological processes, respiratory dysfunction and hypoxaemia caused by the infection may lead myocardial injury. Covid-19 induced hypoxemia can also trigger the development of atrial fibrillation and anticoagulation in the setting of marked inflammation may pose a dilemma [61].

There is emerging evidence that Covid-19-induced hypercoagulability may play a significant role in overall Covid-19 outcomes and there is promising evidence to support the use of therapeutic anticoagulation in high-risk individuals. Aryal et al [62] proposed mechanisms for Covid-19-associated coagulopathy, as well as algorithms for screening, prevention (including extended-duration prophylaxis), and treatment of these patients.

## Impact of medications

Some of the drugs currently being investigated for the treatment of Covid-19 include CQ and HCQ. These agents may increase depolarization length duration and Purkinje fiber refractory period [63]. This may lead to AV node and Bundle of His system dysfunction. In a recent observational study, HCQ administration was not associated with either a greatly lowered or an increased risk of the composite end point of intubation or death in Covid-19 [64].

CQ and HCQ accumulate in lysosomes, inhibit phospholipase activity, induce cytoplasmic inclusion body formation, increase lysosomal pH and cause protein inactivity [65,66]. In view of these properties, these drugs can cause drug-induced atrial and ventricular arrhythmias. The commonest ECG change is fascicular block which may degenerate to advanced AV block leading to syncopal attacks [66]. Hydroxychloroquine also induces prolongation of the QT interval [67]. This side-effect is uncommon but when it occurs, it has the potential of inducing fatal ventricular tachycardia. The mechanism of this is not well understood, however, in one animal study, it was shown that HCQ has an inhibitory effect on ‘the hyperpolarization-activated current ion channels (also known as funny current channels), along with delayed rectifier potassium currents and L-type calcium ion currents’ [60].

This finding is similar to the proposed mechanism by which refractory action potentials in cardiac myocytes may result in the prolongation of the QT interval as a result of delayed depolarization and repolarization from an abnormal ion current [68]. Because QT prolongation is very unpredictable, dose adjustment is required in the use of these medications, especially in individuals with cardiovascular and renal diseases. The pro-arrhythmic risk of the drug should be monitored with 12-lead ECG. Caution should be applied in patients with rhythm abnormalities, electrolyte imbalance and concurrent use of drugs that prolong the QT interval. Although there are concerns about safety of HCQ and CQ, these drugs have been used extensively in Africa in the past for treatment of malaria, with no major safety signals raised [69,70].

Calcium channel blockers (CCB) are common anti-hypertensive medications in Africa [71]. Because they are mainly metabolized by cytochrome P450 3A (CYP3A) isoenzyme, their activity can be significantly inhibited by anti-viral drugs such as ritonavir [72]. Therefore, concomitant treatment must be carefully monitored in clinical practice because of the high risk of side effects [72].

In a study, there is a suggestion that this drug-drug interaction can be adequately managed by adjusting CCB doses and clinically monitoring BP and electrocardiographic assessment, thereby reducing rates of discontinuation of the CCBs or change in antiretroviral regimen.

There is also evidence that SARS CoV requires Ca^2+^ ions for host cell entry [73], and based on the similarity between SARS CoV and SARS CoV 2 [73], it is highly likely that the same requirements exist for the two viruses.

Studies have shown that amlodipine, felodipine and nifedipine limit the growth of SARS CoV 2 in epithelial kidney (Vero E6) and epithelial lung (Calu-3) cells with felodipine and nifedipine having the greatest effect. Overall, evidence suggests that CCBs have a high potential to treat SARS CoV 2 infections and their current FDA approval would allow for a fast repurposing of these drugs [74].

Dexamethasone is another drug used in the management of Covid-19 [75,76,77]. It is said to mitigate the lung injury and multi-organ dysfunction that can occur. No cardiac complications have been ascribed to this medication; however, it can increase the risk of hyperglycemia, secondary infection, psychiatric complications and avascular necrosis [78]. It can also increase the risk of reactivation of latent infections (such as tuberculosis, hepatitis B, herpes infection and strongyloides especially in the African environment where these infections/infestations are common [78]. Furthermore, since dexamethasone is a cytochrome p450(CYP)3A4 inducer, it may reduce the plasma concentration and potential efficacy of drugs that are CYP3A4 substrates [78].

Although there is no formal study to date on the effect of co-administration of dexamethasone and remdesivir, a clinical drug reaction is not predicted.

## Possible long-term effects of Covid-19

Long-term follow-up of individuals who survived Covid-19 shows that about 68% continued to have lipid abnormalities, 40% had cardiovascular disease and altered glucose metabolism was noted in 60% after a 12-year follow up [79].

For now, we do not know the long-term consequences of Covid-19, but because it shares similar biology with SARS, these abnormalities will not be unexpected in the future. This will definitely escalate the rising burden of metabolic and cardiopulmonary diseases in SSA [80,81].

## Management of Covid-19 patient with cardiovascular disease

The management principles are the same for those who do not have cardiovascular disease. Some of the important areas of consideration for a cardiac care health worker include:

1. Personal protection equipment (PPE) while caring for Covid-19 patients. There is need for proper training in donning, usage and doffing of PPE in accordance with the best practices and guidelines.
2. Health systems and hospital preparation. Rapid development and regular rehearsal of guidelines for rapid diagnosis, isolation and management of cases. There should be judicious use of cardiac biomarkers and imaging procedures. The risk-benefit ratio should be assessed in the use of fibrinolysis or primary PCI in Covid-19 patients with acute coronary syndrome. There is no available evidence to discontinue ACEI/ARB in Covid-19 patients. Most guidelines recommend that these medications should not be discontinued. Cardiologists caring for Covid-19 patient should be familiar with the adverse side effects of medications currently used for the management of this disease such as HCQ, antiviral drugs, corticosteroids, etc. Various antiviral drugs also significantly interact with cardiac drugs and will therefore need dose modifications. The major challenge for Africa is that most countries will not be able to provide adequate PPE for healthcare providers.

There may also be challenges for isolation of mild-moderate cases that are not admitted to hospitals in settings with low health literacy and poverty in Africa.

However, many questions remain at the current time. We currently do not have data on whether younger African patients with existing conditions such as rheumatic heart disease, cardiomyopathy or unoperated congenital heart disease have an increased risk of having a profound effect from a Covid-19 infection. The World Heart Federation Survey on Covid-19 and Cardiovascular Disease has commenced in May 2020 and will hopefully provide some answers.

## Broad Impact on African Health Care System

Broadly reflecting their economic status, health systems across many countries in Africa are poorly resourced and underdeveloped. On a more fundamental level, around half of the 783 million people living in Africa do not have access to clean water. This makes hand-washing and personal hygiene practices difficult in many parts of the continent [82]. Both physical and financial access to healthcare is poor. Human resources for health in most parts of Africa is low, and there are inadequate bed spaces to accommodate a monumental pandemic. There is also widespread lack of national plan and preparedness to handle epidemics.

Alternatively, the continent has often faced the challenges of managing infectious disease outbreaks and had to become resilient to such provocations to personal health and the services needed to overcome them. Forty-seven countries making up 87% of the continent has had to grapple with disease outbreaks in recent times. There were outbreaks of Ebola between 2014–2016 in West Africa and between 2018–2020 in the Democratic Republic of Congo. Nigeria is still a ‘hot-bed’ for outbreaks of Lassa fever. On the positive side, these outbreaks have led to some improvement in disease surveillance, epidemic preparedness and clinical and laboratory capacity in the region. Challenges include the availability of diagnostic tools, limited hospital beds, limited medical supplies as well as lack of strategies aimed at flattening the curve.

The budgetary allocation to health care is low in most African countries under the Abuja Agreement of 15% [83]. Tax rates as a percentage of the gross domestic product (GDP) are also low in Africa (23% compared to 53% in North America) The dependence on external funding for health programs is also high. In addition, capital flight from the continent is significantly high as well [84]. Therefore, global financial interdependencies will exert powerful negative effects on Africa’s capacity to address economic impacts of health emergencies [85]. The impact of the current pandemic on the health-related supply chain in Africa may be catastrophic. For instance, the 2014 Ebola epidemic was associated with an estimated economic and social cost of about 53 billion USD [85].

The concept of self-isolation and physical distancing is also a big challenge in Africa. The urban cities in the continent are densely over-populated and the rural areas are over-crowded and have poorly ventilated huts and houses. Health workers are also inadequate to help in the implementation of preventive measures at the community level. Physical distancing may also be difficult to be adhered to in most parts of Africa. The traditional markets and public transports are crowded. The cities are also characterised by traffic jams. Travel restrictions may therefore be a better alternative. However, this may result in social discontent as has been reported in some of the other developing countries outside Africa [86]. These measures are also difficult to enforce among people displaced by conflicts who are in camps around the continent either as internally displaced persons or refugees.

A social and economic ‘lockdown’ approach is useful and has been demonstrated to work in many parts of the world (e.g. Australia and New Zealand) in limiting Covid-19 transmission, health service utilization and related mortality. This, however, may also not work for a long time in Africa in the absence of robust economic packages and social support for the citizens. Furthermore, in the urban slums and rural areas of Africa, there are shared facilities such as water and sanitation facilities. Working from home may also not be possible in many parts of the region because the majority of the populace engages in trading businesses that are not confined in offices.

## Impact on care of HIV/AIDs and other common conditions in Africa

Covid-19 pandemic poses significant threat to people living with HIV/AIDS worldwide, especially in sub-Saharan Africa where the prevalence of the disease is high. About 38 million people live with HIV/AIDS globally and around 33% (12.6 million) are unable to access care. The pandemic is likely to have greater impact on this vulnerable and often marginalized population. Recent data from the WHO and UNAIDS suggest that there could be about half a million extra AIDS-related deaths in sub-Saharan Africa in 2020–2021 if the pandemic results in a six-month disruption in the supply chain for medications for this population [87,88].

Other endemic diseases in Africa, such as tuberculosis and malaria, may also suffer similar fate. Furthermore, the chronic malnutrition common in Africa may lead to worse outcomes for Covid-19 in Africa [88].

## Socioeconomic impact of the Covid-19 pandemic

The rapid evolution of the novel SARS-CoV-2 virus outbreak in Wuhan, China into a pandemic in less than 12 weeks has impacted the global economy with many uncertainties. The world (including Africa) is already faced with a potentially monumental socio-economic crisis as a result of direct and indirect consequences of the pandemic [89,90,91]. As with other pandemics and war-like situations, the future may not be accurately predicted. Several postulations on the socioeconomic impact of Covid-19 have been proposed. In one model, the international monetary fund (IMF) has already projected a global economic recession in 2020 [86,92] and most high-income countries are predicting sharp economic down-turns that exceed the Great Depression. Disruption in the global value chains, the rapid decrease in commodity prices and monetary incomes as well as social and travel restrictions are some reasons for the declining global economy.

With a high rate of transmission, stringent measures to contain the virus need to be implemented. Travel and social restrictions are one of the recommendations to curb the spread of the virus. Although the WHO did not recommend large-scale international travel restrictions or border closure [13], several governments have instituted strict travel and social restrictions, including the lockdown of national and local borders [86,89]. These restrictions put a lot of strain on the supply-and-demand chain of goods and services [93]. Many countries may also downsize their workforce. The global rate of unemployment is projected to increase with about 5.3–24.7 million persons likely to be adversely affected [90].

Similar to other low-to-middle income regions of the world, the relative socioeconomic impact of the Covid-19 pandemic is likely to be worse in Africa compared to higher-income countries worldwide [90]. The growth of the continent’s GDP is projected to fall abysmally from its current annual rate of 3.9% to 0.4% in the best case scenario [85]. If the virus is not contained sooner than later, Africa may experience sustained negative growth in GDP [94]. Sadly, as indicated, the number of confirmed cases of Covid-19 is still rising across Africa notwithstanding travel restrictions in place [93].

Oil is a major source of energy in the manufacturing and transportation sector. As a result of the imposed restrictions on these sectors, the demand for crude oil has been drastically reduced. The result is an unprecedented acute fall in oil prices. Resource-constrained economies are projected to be negatively hit by the oil shock. For instance, the major source of Nigeria’s foreign exchange is through the export of crude oil. As a result of the current global oil shock, it is projected that Nigeria’s GDP growth rate for the year 2020 will drop from 2.5% to 3.4% [86,89].

It is opined that the lockdown in many parts of Africa was premature and hastily applied. With an informal workforce that far outnumbers those in the formal sector, the majority of whom depend on daily wages for subsistence, enforcing a stay-at-home order could have invoked a doomsday scenario for those affected. In the event of non-existent or poorly structured relief packages, the poor are typically stranded, whereas the rich can afford to buy and stockpile foodstuff and other essential products. The lockdown has indeed heightened existing socioeconomic disparities and health inequities even in high-income economies [95,96]. Thankfully, the lockdown is being gradually eased [97,98].

On a positive note, there are possible advantages of the early lockdowns in Africa including the possibility of creating a change of attitudes and practice in high-risk groups.

Opportunities, however, are abound for Africa to grow its economy even in the face of the Covid-19 pandemic [98]. Small and medium scale enterprises, and even national and multinational companies, may need bailouts to get back on track in the post-Covid-19 era. Hitherto, many African countries depend on importation for some staple foods like wheat and rice as well as medicines and machinery. The narrative can change with the implementation of the right policies, and goodwill to diversify the economy and support entrepreneurs.

## Prospects and opportunities

The Covid-19 pandemic presents the opportunity for governments of African countries to strengthen and support the capacity and performance of their hitherto failing health systems. At the initial stage of the Covid-19 pandemic, it was thought that the burden of the disease in Africa would be low. The reasons adduced included the volume of air transport in the region which is lower than most parts of the world. For instance, Gilbert et al used data on the volume of air transport to estimate the risk of importation of the disease to Africa [99]. The authors projected that importation risk would be higher in Algeria, Egypt and South Africa. Moderate risk countries were Angola, Ethiopia, Ghana, Kenya, Sudan and Tanzania. Other reasons include the lack of large-scale testing and reporting, effective border screening and possible vulnerability of the virus due to Africa’s hot climate. The last factor may not be true as there are community transmissions in similar climates in South America and South-East Asia and in many parts of Africa [99].

There is a need to domesticate policies and preventive measures. There is also need for a broad-based effective collaboration to tackle the pandemic in Africa. Herein lies the role of key players in this effort. This includes the World Health Organization AFRO region, African Union Commission, member states, national ministries of health, the African task-force for coronavirus preparedness and response (AFTCOR) the African Development Bank, the African Centre for disease control, national centres for disease control, and the private sector. Consequently, African countries, as a matter of urgency, need to include healthcare in their international policy and engagements. Collaboration in health matters within Africa and without needed to be encouraged. In this regard, Cuba has had success in health diplomacy. African countries may benefit from replicating this strategy.

African Americans also share similar social determinants of health with Africans residing in sub-Saharan Africa apart from high burden of hypertension, type 2 diabetes mellitus, obesity and high prevalence of cardiovascular diseases. Many reside in poor areas characterised by high housing density and poor access to healthy foods and quality of healthcare [100,101].

Studies have shown that African Americans are less likely to maintain social distancing, less likely to work from home and less likely to have access to telecommunication and visual social events. They also have lower Covid-19 knowledge levels, lower intent to comply with control measures as well less access to Covid-19 related information [102,103,104].

Although mortality from Covid-19 is reported to be high for African Americans [105,106], this is not so in native Africans. Some of the reasons adduced to this include quick action by African governments public support, young population and few old peoples’ homes, favourable climates, and good community health systems. Others include the possibility of pre-exiting protective immune responses due to either having had previous exposure to other pathogens or the BCG vaccination [107,108]. Genetic factors may also have a significant role. Haplotype associated with increased risk of severity were found in 30% of South Asian genomes and 8% of Europeans but not in Africans [109].

## Conclusions

Although the acute respiratory complications are common in Covid-19, cardiovascular complications are increasingly being recognized. These include acute myocardial injury, arrhythmias and cardiac failure. Therefore, there is a need for a high index of suspicion of cardiovascular complications since there is overlap in the symptoms and signs. Medication commonly used in the management of the disease also has cardiovascular complications, and these need to be monitored as well.

The broad African economy, though generally growing, remains fragile. Overall, the African health system is fragile and under-resourced. Therefore, the Covid-19 pandemic may have significant negative effects both in the coming months of active cases and the post-pandemic recovery. Rather than consider this as a fait accompli, the very same Covid-19 pandemic/crisis represents the stimulus and opportunity for governments of African countries to strengthen and support the capacity and performance of their hitherto failing health systems. There is critical need to domesticate policies and preventive measures. There is also a need for a broad-based effective collaboration to tackle the Covid-19 pandemic in Africa to mitigate potentially catastrophic health and economic impacts among some of the most vulnerable people and communities in the world.

At the time of writing this paper, a number of vaccines (e.g. BNT162b2/COMIRNATY Tozinameran (INN) by Pfizer, AZD1222 by AstraZeneca, Covishield (ChAdOx1_nCoV19) by Serum Institute of India, SARS-CoV-2 Vaccine (Vero Cell), Inactivated (lnCoV) by SinoPharm/Beijing Bio-Institute of Biological Products Co-Ltd (BIBP) etc) have been approved by the WHO. As at mid-February 2021, there are at least seven different vaccines accros three platfors that have been rolled out in some countries.

Apart from the challenge of determining their efficacy in the light of new strains of the virus (e.g B.1.1.7 in the United Kingdom, B.1.351 in South Africa, and P.1 in Brazil), the issue of equitable access to these vaccines remains a major issue. Based on the current availability, affordability and practical distribution (particularly in regards to “cold-chain” networks) the likelihood of COVID-19 control in Africa will not be achieved soon.
